# RESIDENTIAL HOMES FOR THE DYING: AN UNTAPPED RESOURCE FOR TEACHING PATIENT- AND FAMILY-CENTERED END-OF-LIFE CARE

**DOI:** 10.1093/geroni/igz038.2666

**Published:** 2019-11-08

**Authors:** Chantelle Sharpe, Carol Weisse

**Affiliations:** 1 Connecticut College, New London, Connecticut, United States; 2 Union College, Schenectady, New York, United States

## Abstract

Clinical training opportunities in end-of-life care are lacking, especially in home settings where death is expected and supported as a natural process. The Community Action, Research and Education (CARE) program provides students who are interested in healthcare a better understanding the challenges of providing end-of-life care. Over 8 weeks, undergraduate students serve as surrogate family members providing care to hospice patients in residential homes for the dying. Additionally, students engage with a formal curriculum by completing online learning modules each emphasizing different skills for providing end-of-life care. This study analyzed data from three cohorts of undergraduate students (n = 21) who participated in the CARE Program. Analyses from assessment surveys revealed that students reported improved knowledge and skills, including enhanced bedside education and training and increased ability to care for someone at the end-of-life after completion of the program. Also, 95% (n = 20) of students over the three cohorts reported that the formal coursework enhanced skills and training related to bedside care. Previous research has examined end of life training in a professional school setting, but the focus was on care in an institutional or facility setting (Billings et al., 2010; Supiano, 2013). The CARE program is a model for experiential learning in a home setting that provides a special lens to the dying experience in a holistic, patient and family centered way.

